# Discussion on Syphilis

**Published:** 1906-05-12

**Authors:** 


### DISCUSSION ON SYPHILIS.

On the continuation of the discussion upon syphilis before the Hunterian Society, Dr. Shennan (Edinburgh), who has done a large amount of work on the subject of the organism of syphilis, demonstrated in a very striking manner the spirochaete pallida as being the specific organism. He briefly reviewed the work that had been done on the subject by the various workers. He then pointed out how difficult it was to demonstrate the organism, and said it was well that there should always be on the preparation some such thing as blood-corpuscles, in order that there be something definite to focus. He came to the conclusion that this spirochaete pallida was the organism on account of the following facts:—(1) That it was found in non-ulcerative lesions; (2) its presence in circulating blood; (3) its presence in syphilitic infants; (4) its presence in primary and secondary eruptions, and never in non-syphilitic eruptions. Against the organism as being the syphilitic one, he mentioned the following facts:—(1) Scanty number found; (2) that there are intermediate forms; (3) that similar forms had been found in other lesions. The spirochæte was mos tcommonly found in the liver, lung, suprarenals, and skin; in the spleen and kidneys not so common. The liver is the organ to be first affected. If the organisms are scanty then they are generally found near blood-vessels. With regard to the degeneration of the organism there was a difference of opinion as to when this took place, some saying that it occurred in six to seven hours, while others say it keeps much longer.

Dr. Shennan exhibited a most beautiful collection of slides, showing the organism in various stages, and also demonstrating organisms very like the spirochaete.

"Syphilis as met with in Children" was dealt with by Dr. G. F. Still. The acquired form, he said, was very rare indeed, the inherited form being the common one in children. Contagion from the inherited form he considered as very exceptional. If it did occur it would be through such sources as nasal discharges, fissures on the lips, etc. A very important point, Dr. Still remarked, was the fact that about 10 per cent. of those suffering from inherited syphilis developed some nervous trouble—*e.g.* idiocy, progressive mental deterioration, hydrocephalus, etc. Convulsions also could often be traced to a syphilitic origin. There was a marked tendency for certain tissues to be affected in certain families.

The importance of syphilis as an ætiological factor in the production of interstitial nephritis in children was touched upon. With regard to bone and joint affections, Dr. Still queried the saying that syphilis was a cause of cranio-tabes, as syphilis usually produced a thickening of bone. Joint affections were fairly common, and of these there were two varieties—(1) A simple synovitis, single or multiple; (2) an osteo-arthritis, occurring in the second half of childhood, and responding very little to treatment.

Then Dr. Still pointed out the extreme importance of examining the eyes, as, in his experience, at least 21 per cent. show ocular changes.

With regard to treatment, he said there was nothing like grey powder, in small doses, three times a day; or, if the child would not take a powder, then a dose of the liq. hydrarg. perchlor. (4-10 m., according to age) should be given.

Treatment must be continuous over a period of at least two years after the disappearance of all symptoms.

The subject of "Syphilitic Affections of the Skin" was introduced by Dr. Morgan Dockrell, who showed an extremely beautiful collection of illustrations of syphilitic skin affections.

He pointed out the importance of remembering that when an ordinary skin eruption does not respond to recognised treatment, that then anti-syphilitic remedies should be tried, and the result would in many cases prove the true nature of the affection. It was also, he said, necessary to remember that skin eruptions occurring on a syphilitic base should be treated locally as well as by the internal administration of anti-syphilitic remedies.

Dr. J. McCulloch Ettles discussed the general treatment of syphilis. He pointed out that as soon as the disease was recognised, mercury should be administered, as this drug had a very marked effect in lessening the severity of the secondary symptoms and in preventing the tertiary manifestations. He laid great stress on the value of mouth administration as compared with inunction. Only a very small proportion (about 1 in 200) was absorbed through the skin, and the method was dirty, tedious, and uncertain. Intramuscular injections were very useful in certain cases, but great care was needed in using these to see that the injections were made into a muscle, and that the persons injected were active and muscular people. For injections he regarded Lambkin's cream of finely divided metallic mercury, suspended in an oily menstruum, as the best preparation to use.

The value of general hygienic measures could hardly be exaggerated. Syphilis was always much less troublesome in robust men than in weakly individuals. The treatment should be continuous, and not intermittent, and should be kept up for at least *two* years after the disappearance of sypmtoms. If these symptoms were tertiary, either short courses of iodides should be kept up for five or six years after, as suggested by Sir William Gowers, or the patient should for the rest of his days take small doses of mercury. The iodides were of enormous value in causing the disappearance of gummata, but it appeared that mercury was needed to abolish the specificity. Dr. Ettles also alluded to the value of Zittmann's decoction.

The question of the treatment of syphilis in cases of Bright's disease was raised. Such cases could not stand mercury well, and he had obtained the best results by giving 8½ grains of iodide of iron in the form of two drachms of fresh syrup of iodide three times daily in large quantities of water.

A letter was then communicated by Dr. Fortescue Fox which he had received from Dr. Francon, Aix-les-Bains, and in which the treatment of syphilis by inunction was described, and the great value that he (Dr. Francon) attached to it. Also the fact that recent experiments had proved the value of sulphur water taken in combination with the mercurial treatment as greatly assisting the action of the mercury.

In conclusion, Dr. F. J. Smith (President), who was in the chair, congratulated the Society on the success of its meetings on the question of "Syphilis." He thanked, in the name of the Society, those gentlemen who had so ably introduced the various subjects under discussion. He regretted much that time would not permit of any further discussion, but he felt that everyone who had attended the meetings would go away with a storehouse of knowledge on the question of syphilis.

